# Trends in Vaccination Coverage among Children Aged 2–6 Years in Tennessee Counties, 2017–2023

**DOI:** 10.3390/vaccines12091048

**Published:** 2024-09-13

**Authors:** Walid Q. Alali, Qian Huang, Kate Goodin, Adrian Gonzalez-Lozano

**Affiliations:** 1 Department of Biostatistics & Epidemiology, College of Public Health, East Tennessee State University, Johnson City, TN 37614, USA; huangq@etsu.edu (Q.H.); kate.goodin@tn.gov (K.G.); 2 Center for Rural Health Research, College of Public Health, East Tennessee State University, Johnson City, TN 37604, USA; 3 Surveillance Systems and Informatics Program (SSIP), Communicable & Environmental Disease & Emergency Preparedness (CEDEP), Tennessee Department of Health, Nashville, TN 37243, USA; 4 Vaccine-Preventable Diseases & Immunization Program (VPDIP), Communicable & Environmental Disease & Emergency Preparedness (CEDEP), Tennessee Department of Health, Nashville, TN 37243, USA; adrian.gonzalez-lozano@tn.gov

**Keywords:** immunization, pediatrics, COVID-19, pandemic, vaccination

## Abstract

**Background/Objectives**: This study examines trends in county-level vaccination coverage before, during, and after the COVID-19 pandemic among children aged 2–3 and 4–6 years in Tennessee, with a focus on rurality; **Methods**: Data from the Tennessee Immunization Information System (January 2017 to September 2023) were analyzed for vaccination coverage in children in both age groups. The study categorized the COVID-19 pandemic into three periods: pre-pandemic (P1: January 2017 to December 2019), stay-at-home (P2: January 2020 to May 2021), and reopening (P3: June 2021 to September 2023). Vaccination trends were stratified by vaccine type, rurality, sex, race and ethnicity; **Results**: During P1, there were no significant changes in trends of vaccination coverage percentages in both rural and urban counties for both age groups. However, vaccination coverage declined significantly during P2 and P3 compared to P1 for most vaccines, except for influenza, which initially increased but later declined. Rural counties experienced a more pronounced decline compared to urban counties during P2 and P3 for both age groups. Within rural and urban counties, vaccination coverage was higher among white children compared to black children, and among non-Hispanic compared to Hispanic children. There were higher coverage percentages in age group 4–6 for all vaccines, except for influenza, compared to 2–3 year group; **Conclusions**: The COVID-19 pandemic has exacerbated disparities in childhood vaccination coverage, particularly in rural areas. These findings highlight the need for targeted public health interventions to address barriers to vaccination and ensure equitable access to vaccines for all children.

## 1. Introduction

The COVID-19 pandemic has dramatically reshaped the landscape of public health, presenting unprecedented challenges and complexities for healthcare systems worldwide [1]. Amidst the urgency of combating the novel coronavirus, maintaining routine healthcare services, particularly childhood immunization, has emerged as a critical concern [2]. Childhood immunization stands as one of the most effective public health interventions, preventing the spread of infectious diseases and safeguarding the health of children and communities through herd immunity [3]. The pandemic has disrupted healthcare delivery systems, interpreted education systems, strained resources, fueled vaccine hesitancy, and exacerbated existing barriers to immunization access and uptake [4,5].

Trends in vaccination coverage in relation to sociodemographic factors such as race, ethnicity, education, and income have been extensively studied at the individual level [6,7,8,9]. However, there are limited data available on trends in vaccination coverage by rurality (rural vs. urban areas) and in relationship to the COVID-19 pandemic at the community or county-level. Various reports have shown that the pandemic had a greater impact on the healthcare systems in rural areas where resources are scarce compared to urban areas [10,11,12,13,14,15,16]. For instance, access to primary healthcare providers in rural areas was impacted due to the pandemic, which in turn might reduce childhood vaccination rates and likely increase the incidence of vaccine-preventable diseases (VPDs) [17,18,19]. A study showed a 5% decline (2019 vs. 2020) across all child required vaccines in 24-month-old children in Texas, with a larger decline observed in rural areas compared to urban areas [17]. However, their study was conducted during 2020 (second year of pandemic) and on children less than 24 months. Therefore, trends in vaccination coverage were not assessed in the following years. Similarly, vaccination rates in multiple states were significantly lower by September 2020 compared to previous years in children under 24 months old, particularly among non-hispanic black children [20]. Therefore, it is imperative to demonstrate trends in vaccination coverage in children over the course of the pandemic at the local/regional level and by potential contributing factors to inform public health efforts.

This study examined trends in county-level up-to-date (UTD) vaccination coverage among children aged 2–3 and 4–6 years in Tennessee between January 2017 and September 2023 before, during, and after COVID-19 pandemic. Furthermore, we assessed vaccination coverage trends by rurality, sex, race and ethnicity at the county-level based on available data.

## 2. Materials and Methods

### 2.1. Study Population and Data Sources

The vaccination coverage rates for children ages 2–3 years and 4–6 years who are UTD on their recommended vaccinations by that age range according to the Advisory Committee on Immunization Practices (ACIP) schedule vaccines [21] were included in this study. Estimated county-level data for both age groups were obtained from the Tennessee Immunization Information System (TennIIS) Registry, encompassing before, during, and post COVID-19 pandemic in Tennessee. TennIIS is an opt-in registry managed by the Tennessee Department of Health (TDH) that obtains and stores vaccine records from different sources, including health care providers, pharmacists, schools, and childcare administration [22]. For the purposes of this study and to protect patient health information (PHI), analyses were performed at the county level. This data was then used in conjunction with U.S. census population data, which provides detailed counts of individuals in each county. Aggregate counts of UTD vaccinated individuals were first obtained from TennIIS, and then divided by the total population of each county annually to calculate vaccination coverage percentages.

The vaccination coverage percentage data were extracted from TennIIS in December 2023. The percentages represented county-level estimates for the period between January 2017 and September 2023 (approximately 7 years) for two age groups: 2 to 3 years and 4 to 6 years. The monthly vaccination coverage percentages for children who completed the required dosage (as shown in Table 1) were recorded for each county in Tennessee and for both age groups. County-level variables in the datasets included vaccine name, county name, biological sex (male and female), and race (white and black) and ethnicity (hispanics and non-hispanics). Based on the extracted available data, we created two additional variables: (1) COVID pandemic categorized into three periods based on Tennessee’s COVID timeline [23] as follows: Pre-pandemic period (P1: January 2017 to December 2019), stay-at-home period (P2: January 2020 to May 2021), and reopening period (P3: June 2021 to September 2023), and (2) rurality where counties were classified as either rural or urban based on the 2023 Rural-Urban Continuum Codes (RUCC)-Economic Research Service-United State Department of Agriculture (USDA) [24].

Due to an unforeseen error in the TennIIS system’s forecaster, Hepatitis B vaccination coverage for 4–6 year age group were inaccurately evaluated and assessed, resulting in percentages slightly exceeding 100%. As such, the percentages were excluded to maintain the integrity and accuracy of the data presented.

### 2.2. Data Analysis

Summary statistics of UTD vaccination coverage percentages, including means and 95% confidence intervals (95% CI), stratified by the study variables, were calculated and presented in tables and bar graphs. Differences in trends in vaccination coverage percentages were evaluated and compared by COVID pandemic periods, rurality, sex, race and ethnicity using non-parametric Kruskal-Wallis test in STATA version 17.0 (StataCorp LLC, College Station, TX, USA).

## 3. Results

**Figure 1:**
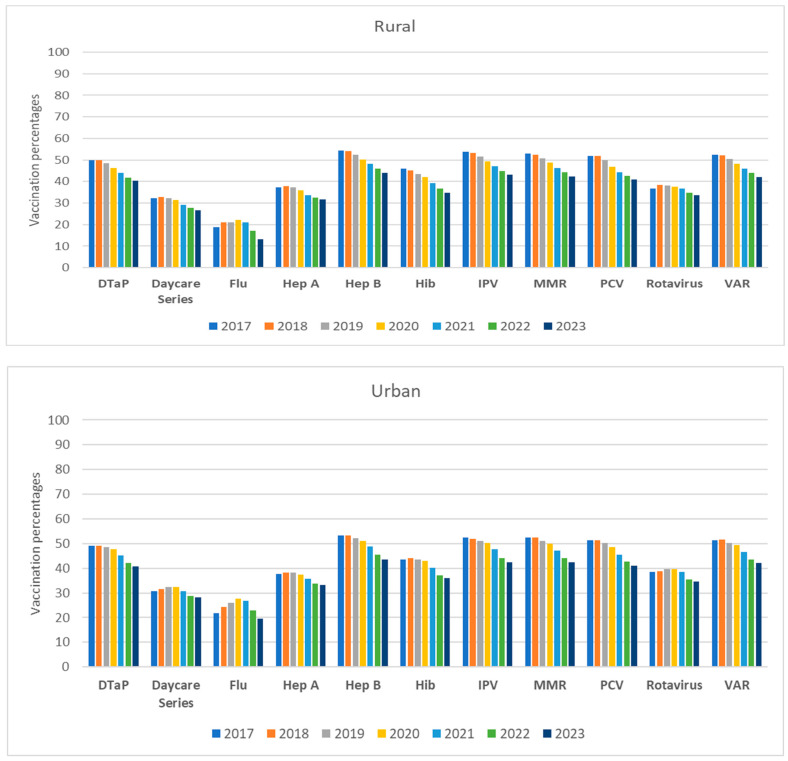
Trends in percentage of vaccine coverage at county-level for 2–3 years age group stratified by vaccine type, rurality, and year in Tennessee (2017–2023).

**Figure 2:**
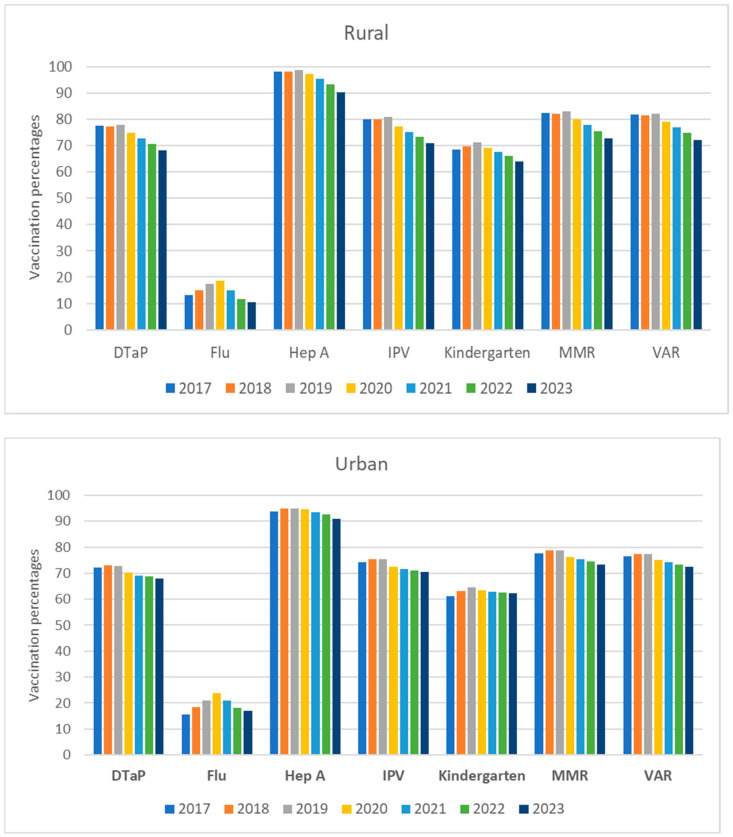
Trends in percentage of vaccine coverage at county-level for 4–6 years age group stratified by vaccine type, rurality, and year in Tennessee (2017–2023).

The overall UTD vaccine coverage percentages and their 95% CI stratified by vaccine, rurality, and COVID-19 pandemic are shown in Table 2 and Table 3 as well as in Figure 1 and Figure 2. Overall, trends in vaccination coverage for age groups 2–3 and 4–6 years declined significantly during both periods P2 and P3 compared to P1 for all vaccines except for influenza. For influenza, the peak for both age groups occurred during the pandemic in 2020, followed by a steep decline in 2021 through 2023. Coverage percentages for all vaccines were higher in the 4–6 age group compared to the 2–3 age group, except for influenza. Although it is recommended that the two doses of the Hepatitis A vaccine be completed by age three, we observed significantly higher coverage in the 4–6 age group which may indicate a delay in being UTD on Hepatitis A vaccine in children in Tennessee during the study period.

### 3.1. Age Group 2–3 Years

During the pre-pandemic period (P1: 2017–2019), there were no significant changes in trends of UTD vaccination coverage percentages in both rural and urban counties except for influenza. Interestingly, the largest increase was by about 11%, which occurred between 2017 and 2018 for influenza coverage in both rural and urban counties (Table 2 and Figure 1), followed by a significant decline.

During the P2 and P3 COVID-19 pandemic periods, trends in vaccination coverage percentages in this age group declined significantly compared to P1 for almost all vaccines. Vaccination coverage decreased during the first year of the pandemic and remained reduced as of September 2023, with a significantly higher decline in rural counties compared to urban counties (Table 2 and Figure 1). For instance, the daycare vaccine series coverage in rural counties declined by 4.6% during P2 compared to P1 (*p* < 0.05) and 10.4% during P3 compared to P2 (*p* < 0.05). In urban counties, the daycare vaccine series significantly declined by 1.6% (P2 vs. P1) and 9.8% (P3 vs. P2) (Table 2).

When comparing individual vaccine coverage between P2 and P1 as well as between P3 and P2 in rural counties, the largest change was for influenza vaccine (increased by 9.3% and then decreased by 25%, respectively) and rotavirus (decreased by 0.6% and then by 7.2%, respectively). Similarly, in urban counties during the same periods, the largest change was for influenza coverage (increased by 15.3% and then decreased by 18.6%, respectively) and rotavirus (increased by 1.4% and then by 9.2%, respectively).

There were no significant differences in vaccination coverage percentages by sex within the rural counties for all vaccines. However, coverage percentages were significantly different by race, with whites having higher coverage than blacks. Moreover, non-Hispanics had significantly higher coverage than Hispanics within rural counties. Similarly, within urban counties, there were no significant differences in vaccination coverage by sex for all vaccines. Coverage percentages were significantly different by race, with whites having higher coverage than blacks for all vaccines. Non-Hispanics had higher vaccine coverage than Hispanics within urban counties for all vaccines.

When comparing vaccination coverage percentages *between* rural and urban counties by sex, race, and ethnicity, there were no differences by sex. Whites in urban counties had higher coverage percentages than whites in rural counties for DTaP, daycare series, Hep B, HiB, IPV, MMR, and VAR, but lower for the flu. Blacks residing in rural counties had no significant difference in vaccination coverage than those residing in urban counties, except for the flu vaccine, which was higher in urban counties (Table 2). Hispanics in urban counties had significantly higher vaccination coverage than those in rural counties for all vaccines; however, non-Hispanics in urban counties had no significant difference in vaccination coverage than those in rural counties, except for the flu vaccine, which was higher in urban counties.

### 3.2. Age Group 4–6 Years

During the pre-pandemic period (P1: 2017–2019), there were no significant changes in vaccine coverage trends in both rural and urban counties, except for a consistent increase in influenza vaccine coverage (Figure 2).

During the P2 and P3 COVID-19 pandemic periods, overall trends in the vaccination coverage percentages in this age group (4–6 years) declined significantly for almost all vaccines in both rural and urban counties compared to P1. Vaccination coverage decreased during the first year of the pandemic and continued to decline as of September 2023, with a significantly higher decline in rural counties compared to urban counties for kindergarten vaccine series and influenza vaccination coverage (Table 3 and Figure 2). The kindergarten vaccine series coverage in rural counties declined by 1.8% during P2 compared to P1 (*p* < 0.05) and 3.7% during P3 compared to P2 (*p* < 0.05). However, in urban counties, the kindergarten vaccine series declined insignificantly by 0.1% (P2 vs. P1) and 0.2% (P3 vs. P2) (Table 3).

When comparing individual vaccine coverage in P2 and P1 as well as P3 and P2 in rural counties, the largest change was for influenza vaccine (increased by 17.1% and then decreased by 33.3%, respectively). All other vaccines decreased by about 4–5% over both periods in rural counties. In urban counties during the same periods, the largest change was for influenza (increased by 26.7% and then decreased by 21.7%, respectively). All other vaccines decreased by 3–4% for P2 vs. P1 period and 1–2% for P3 vs. P2 in urban counties.

There were no significant differences in vaccination coverage percentages by sex (male vs. female) within rural counties. Moreover, coverage percentages were not significantly different, with blacks having significantly higher vaccination coverage than whites for DTaP and influenza. Non-Hispanics had significantly higher vaccination coverage than Hispanics for all vaccines except for influenza. Similarly, within urban counties, there were no differences in vaccination coverage percentages by sex for all vaccines. Vaccination coverage was significantly higher for whites than blacks for all vaccines except for influenza. Non-Hispanics had significantly higher vaccination coverage than Hispanics for Hep A, IPV, kindergarten series, and VAR, but lower coverage for influenza. There were no significant differences by ethnicity for DTaP and MMR (Table 3).

When comparing vaccination coverage percentages by sex between rural and urban counties, the coverage for all vaccines for males in urban counties was significantly higher compared to males in rural counties, except for influenza vaccine which was higher in rural counties. Similarly, coverage percentages were significantly higher for all vaccines in females in urban counties compared to females in rural counties, except for influenza which was higher in rural counties. For race, whites in urban counties had higher coverage percentages for all vaccines than those in rural counties, except for influenza which was higher for whites in rural counties. Moreover, blacks in urban counties had higher coverage percentages for Hep A, kindergarten series, MMR, and VAR than those in rural counties. Trends in influenza vaccination among black children in both urban and rural areas were low during the study period. Hispanics had significantly higher vaccination coverage percentages in urban counties for all vaccines compared to those in rural counties. Moreover, non-Hispanics had significantly higher vaccination coverage percentages in urban counties for all vaccines except influenza compared to those in rural counties (Table 3).

## 4. Discussion

The trends in vaccination coverage percentages observed in this study aligns with the findings from multiple studies examining vaccine uptake in relation to COVID-19 pandemic [10,11,12,13,14,15,16]. Our study, which assessed vaccination coverage in children aged 2–3 and 4–6 years in Tennessee from January 2017 to September 2023, found significant declines in vaccination coverage percentages over time during the pandemic, particularly in rural areas. These trends are consistent with the broader literature, which documents similar disruptions in routine healthcare services and immunization programs globally due to the pandemic [1,4].

Several studies have highlighted the disproportionate impact of the pandemic on rural healthcare systems. For instance, access to primary healthcare providers was significantly hampered in rural areas, leading to reduced childhood vaccination rates and an increased risk of vaccine-preventable diseases [17,18,19]. Our findings corroborate this, showing a larger decline in vaccination coverage in rural counties compared to urban ones. For example, the daycare vaccine series coverage in rural counties declined by 4.6% during the stay-at-home period, whereas the decline in urban counties was 1.6%.

The significant decline in vaccination coverage during the stay-at-home and reopening period in Tennessee across most vaccines was reported in this study. The CDC reported that national coverage among kindergartners for DTaP, MMR, polio, and VAR declined by an average of 2% in the 2022–2023 school year [25]. A similar decline was observed in our study (2.2%) for kindergartners vaccine series. Though, the CDC national estimates of vaccine/immunization coverage rates are based the NIS (National Immunization Survey) Child random-digit-dialed survey of household, which is different than our county-level vaccination data. Some major limitations of the NIS include the low household interview response rate, especially during the pandemic, and the availability of adequate health provider data for about 50% of those who complete survey interviews [26]. Our study was based on non-survey vaccine records from different sources across the state, including health care providers, pharmacists, schools, and childcare administration.

Influenza vaccination coverage initially increased during the pandemic, possibly due to heightened public awareness of respiratory diseases, but subsequently declined. This trend was observed in both rural and urban settings, though the magnitude of change was more pronounced in rural areas. Fogel et al. [27], reported that early influenza vaccine rates among children (6 months–17 years) during 2020 were lower than in prior years, which is different than our findings. Their study was based on individual medical records from four medical sites in Pennsylvania and covered a larger age group compared to our study, which was based on county estimates for two age groups across Tennessee.

The literature also points to variations in vaccination coverage by sociodemographic factors such as sex, race and ethnicity [20,28]. In general, our study observed no significant differences by sex within and between rural and urban counties for the 2–3 year age group. White children had higher coverage than black children within both rural and urban counties. When comparing between rural and urban, white children in urban counties had higher coverage for multiple vaccines than white children in rural counties, which agrees with published literature [20,28]. There were no significant differences in vaccine coverage among black children in rural compared to urban counties. In term of ethnicity, non-Hispanics had higher coverage than Hispanics within rural and urban counties. Furthermore, Hispanic children in urban counties had higher vaccination coverage than Hispanic children in rural counties, which is expected and agrees with previous studies [28].

For the older children age group (4–6), while no significant differences were observed by sex within either rural or urban counties, we showed higher coverage among male children in urban counties than those in rural counties, with a similar trend existed for females. Within urban counties, white children had higher vaccination coverage than black children, which was not the case within rural counties. Moreover, white children in urban counties had higher vaccination coverage than white children in rural counties, similar to that in the younger age group. A similar observation was reported for black children. Non-Hispanic children had higher vaccination coverage in rural counties than urban counties; however, for Hispanic children, it was the opposite. Racial and ethnic disparities in pediatric vaccine coverage have widened during the pandemic. Black and Hispanic communities, which have been disproportionately affected by COVID-19, also experienced greater disruptions in routine vaccination services. Research by Patel Murthy et al. [29] highlighted that these communities faced higher rates of missed vaccination appointments due to fear of COVID-19 exposure, limited transportation options, and healthcare facility closures in their neighborhoods. Additionally, urban areas with high COVID-19 case rates saw healthcare resources diverted from routine vaccination to pandemic response efforts, leading to decreased vaccination rates among children.

There are few limitations in this study. First, the vaccination coverage percentages reported in this study might not represent the ‘true’ vaccination status of individuals in Tennessee or those reported by the immunization surveys conducted by TennIIS or the CDC [22,30]. This discrepancy arises due to how individuals living in Tennessee identify themselves in TennIIS versus how they are identified in the U.S. Census population data when calculating coverage percentages. For instance, the discrepancy between the vaccination coverage in P1, P2, and P3 phases and sex coverage are due to how individuals identified their ‘sex’ in TennIIS versus how they were identified in the U.S. Census population data when calculating coverage percentages. This resulted in coverage percentages got inflated for males and females. Therefore, this study assesses trends in vaccination coverage, compares county populations during the pandemic and by rurality, and could potentially measure impact of interventions in the state. However, it should not be compared to the ‘true’ vaccination status of individuals. Second, TennIIS reporting might not capture all vaccine records within the state, including differences in site-based reporting, which might have introduced bias into the vaccine coverage percentages by sex, race and ethnicity.

## 5. Conclusions

Our findings emphasize the critical need to address the barriers to vaccination that were intensified by the COVID-19 pandemic, especially in rural and underserved communities. These results underscore the importance of targeted public health interventions to restore and improve vaccination coverage, ensuring equitable access to vaccines for all children regardless of their geographical or sociodemographic background.

## Figures and Tables

**Table 1:** ACIP list of diseases to prevent through vaccination of children <6 years of age.

Vaccine Name	Vaccine Preventable Disease(s)	No. of Doses (2–3 Year) ^1^	No. of Doses (4–6 Year) ^2^	Possible Complications of Disease
DTaP	Diphtheria, Tetanus, and Pertussis	4 doses	5 doses	Diphtheria: Upper airway obstruction, pneumonia, respiratory failure, and death. Tetanus: Spasms of respiratory and skeletal muscles, death Pertussis: Severe, long-term cough, vomiting, breathlessness, death in infants
Flu	Influenza	At least one	At least one	Secondary pneumonia, exacerbation of chronic diseases, hospitalization, death
Hep A	Hepatitis A	2 doses	2 doses	Fever, nausea, jaundice, death
Hep B	Hepatitis B	3 doses	3 doses	Fulminant hepatitis, jaundice, liver cancer, cirrhosis, premature death
Hib	Haemophilus influenzae type B	3–4 doses	3–4 doses	Pneumonia, meningitis, neurologic problems, death
IPV	Poliomyelitis	3 doses	4 doses	Paralysis, death
MMR	Measles, Mumps, and Rubella	1 dose	2 doses	Measles: ear infections, pneumonia, cardiac and neurologic problems, encephalitis, death Mumps: decreased fertility, meningitis, arthritis, hearing impairment Rubella: arthritis, encephalitis, birth defect
PCV	Pneumococcus	4 doses	4 doses	Ear infections, pneumonia, meningitis, blood stream infections, death
Rotavirus	Rotavirus	2–3 doses	2–3 doses	Dehydration, hospitalization, death
VAR	Varicella/Chickenpox	1 dose	2 doses	Rash illness, severe disease in immunocompromised, birth defects, encephalitis, death

^1^ Daycare Series was part of TennIIS vaccine coverage data collection for children within age group 2–3 years. This series is comprised of the DTaP, IPV, MMR, HBV, Hep A/B, Hib, VAR, and PCV vaccines, and denotes the list of vaccines required of children before they enter the daycare system. ^2^ Kindergarten Series was part of TennIIS vaccine coverage data collection for children within age group 4–6 years. This series is comprised of the DTaP, IPV, MMR, Hep A/B, and VAR, and focuses on the list of vaccines required for children before they enter Kindergarten.

**Table 2:** Percentages of vaccination coverage at county-level for 2–3 years age group stratified by vaccine type, rurality, and COVID pandemic in Tennessee (2017–2023).

	Average Vaccine Coverage and 95% CI																										
Vaccine Name ^1^	COVID Pandemic Periods ^2^									Sex						Race/Ethnicity											
**Rural counties ^3^**	*P1*	*95% CI*		*P2*	*95% CI*		*P3*	*95% CI*		*Male*	*95% CI*		*Female*	*95% CI*		*White*	*95% CI*		*Black*	*95% CI*		*Hispanic*	*95% CI*		*Non-Hispanic*	*95% CI*	
DTaP	49.4	49.0	49.8	45.9	45.4	46.4	41.8	41.4	42.1	45.8	45.5	46.1	46.3	46.0	46.5	42.8	42.5	43.1	43.0	42.3	43.7	45.0	44.5	45.5	46.3	46.1	46.6
Daycare Series	32.3	32.0	32.6	30.9	30.4	31.3	27.6	27.3	28.0	30.4	30.1	30.6	30.5	30.2	30.7	28.8	28.6	29.0	26.9	26.4	27.4	28.1	27.8	28.5	30.8	30.5	31.0
Flu	20.3	20.0	20.5	22.1	21.8	22.5	16.6	16.3	16.9	19.5	19.3	19.7	19.3	19.1	19.5	23.3	23.0	23.5	16.0	15.5	16.5	26.0	25.6	26.4	19.4	19.2	19.6
Hep A	37.5	37.2	37.8	35.3	34.8	35.7	32.4	32.1	32.8	35.2	35.0	35.4	35.4	35.2	35.6	34.1	33.9	34.3	31.7	31.2	32.2	32.8	32.4	33.2	35.7	35.5	35.9
Hep B	53.6	53.1	54.0	49.7	49.2	50.3	45.7	45.4	46.1	49.9	49.6	50.2	50.3	50.0	50.6	46.4	46.1	46.7	45.9	45.2	46.6	49.2	48.6	49.7	50.4	50.1	50.6
Hib	44.8	44.5	45.2	41.4	40.8	41.9	36.6	36.2	36.9	41.2	40.9	41.5	41.3	41.0	41.6	38.4	38.1	38.6	36.8	36.2	37.4	41.2	40.7	41.7	41.5	41.2	41.7
IPV	52.8	52.4	53.2	48.9	48.3	49.4	44.8	44.4	45.1	49.0	48.7	49.3	49.4	49.2	49.7	45.5	45.2	45.8	44.6	43.9	45.2	48.3	47.8	48.9	49.5	49.2	49.8
MMR	52.1	51.7	52.5	48.3	47.8	48.8	44.1	43.7	44.4	48.5	48.2	48.8	48.6	48.3	48.9	45.3	45.0	45.5	44.8	44.1	45.4	47.6	47.1	48.2	48.8	48.6	49.1
PCV	51.2	50.8	51.6	46.3	45.8	46.8	42.3	42.0	42.7	47.1	46.8	47.3	47.2	46.9	47.5	44.2	44.0	44.5	43.9	43.3	44.6	44.7	44.2	45.2	47.5	47.3	47.8
Rotavirus	37.8	37.4	38.1	37.5	37.1	38.0	34.8	34.5	35.1	36.4	36.2	36.7	37.0	36.8	37.3	35.8	35.6	36.1	32.9	32.3	33.5	36.1	35.6	36.5	37.0	36.8	37.2
VAR	51.6	51.2	52.0	47.8	47.3	48.3	43.7	43.4	44.1	48.0	47.8	48.3	48.1	47.9	48.4	44.6	44.4	44.9	44.3	43.7	45.0	43.7	43.2	44.2	48.6	48.4	48.9
**Urban Counties ^3^**	*P1*	*95% CI*		*P2*	*95% CI*		*P3*	*95% CI*		*Male*	*95% CI*		*Female*	*95% CI*		*White*	*95% CI*		*Black*	*95% CI*		*Hispanic*	*95% CI*		*Non-Hispanic*	*95% CI*	
DTaP	48.9	48.5	49.3	47.3	46.8	47.8	42.3	41.9	42.6	46.3	46.0	46.6	46.3	46.0	46.5	44.4	44.1	44.6	42.1	41.4	42.9	41.2	40.0	42.4	46.2	45.9	46.4
Daycare Series	31.6	31.2	31.9	32.1	31.6	32.5	28.9	28.6	29.2	30.8	30.6	31.1	30.7	30.4	30.9	29.6	29.3	29.8	26.1	25.6	26.7	24.5	23.9	25.2	30.9	30.6	31.1
Flu	24.0	23.7	24.4	27.7	27.3	28.2	22.6	22.2	23.0	24.4	24.2	24.7	24.2	23.9	24.4	19.1	18.9	19.3	19.1	18.7	19.5	19.0	18.4	19.5	24.0	23.7	24.2
Hep A	38.0	37.7	38.3	37.1	36.6	37.5	34.0	33.6	34.3	36.5	36.3	36.7	36.3	36.1	36.6	34.3	34.1	34.5	31.4	30.7	32.1	29.4	28.4	30.3	36.6	36.4	36.9
Hep B	52.9	52.5	53.3	50.7	50.2	51.2	45.5	45.1	45.9	49.9	49.6	50.2	49.9	49.6	50.2	48.6	48.3	48.8	45.5	44.7	46.3	44.8	43.6	45.9	49.7	49.5	50.0
Hib	43.7	43.3	44.1	42.5	42.0	43.0	37.4	37.0	37.8	41.3	41.1	41.6	41.2	40.9	41.5	40.0	39.7	40.2	36.9	36.2	37.6	37.0	36.0	38.1	41.1	40.8	41.3
IPV	51.9	51.5	52.3	49.7	49.2	50.2	44.3	43.9	44.7	48.8	48.6	49.1	48.8	48.5	49.1	47.8	47.5	48.0	44.9	44.0	45.7	44.4	43.2	45.6	48.6	48.4	48.9
MMR	51.9	51.5	52.3	49.4	49.0	49.9	44.1	43.8	44.5	48.8	48.5	49.0	48.7	48.4	48.9	47.0	46.8	47.3	44.4	43.6	45.2	43.3	42.1	44.5	48.6	48.3	48.8
PCV	51.0	50.7	51.4	48.0	47.5	48.4	42.7	42.3	43.1	47.6	47.3	47.9	47.4	47.2	47.7	45.7	45.4	46.0	43.0	42.2	43.8	41.0	39.8	42.1	47.6	47.3	47.8
Rotavirus	38.9	38.6	39.3	39.5	39.0	39.9	35.8	35.5	36.2	38.0	37.7	38.2	38.0	37.8	38.2	35.8	35.6	36.0	31.8	31.2	32.4	32.4	31.4	33.4	38.0	37.7	38.2
VAR	51.0	50.7	51.4	48.8	48.3	49.2	43.7	43.4	44.1	48.1	47.9	48.4	48.0	47.7	48.2	46.6	46.3	46.8	44.4	43.6	45.2	40.4	39.2	41.6	48.3	48.0	48.6

^1^ DTaP (Diphtheria, Tetanus, and Pertussis), Flu (Influenza), Hep A (Hepatitis A), Hep B (Hepatitis B), Hib (Haemophilus influenzae type B), IPV (Poliomyelitis), MMR (Measles, Mumps, and Rubella), PCV (Pneumococcus), Rotavirus, VAR (Varicella/Chickenpox). Daycare Series was part of TennIIS vaccine coverage data collection for children within age group 2–3 years. This series is comprised of the DTaP, IPV, MMR, HBV, Hep A/B, Hib, VAR, and PCV vaccines, and denotes the list of vaccines required of children before they enter the daycare system. ^2^ Three COVID pandemic periods were created based on Tennessee’s COVID timeline as follows: Pre-pandemic period (P1: January 2017 to December 2019), stay-at-home period (P2: January 2020 to May 2021), and reopening period (P3: June 2021 to September 2023). ^3^ Rurality where each county was classified as either rural or urban was based on the 2023 USDA-RUCC.

**Table 3:** Percentages of vaccination coverage at county-level for 4–6 years age group stratified by vaccine type, rurality, and COVID pandemic in Tennessee (2017–2023).

Average Vaccine Coverage and 95% CI																											
Vaccine Name ^1^	COVID Pandemic Periods ^2^									Sex						Race/Ethnicity											
**Rural counties ^3^**	*P1*	*95% CI*		*P2*	*95% CI*		*P3*	*95% CI*		*Male*	*95% CI*		*Female*	*95% CI*		*White*	*95% CI*		*Black*	*95% CI*		*Hispanic*	*95% CI*		*Non-Hispanic*	*95% CI*	
DTaP	77.6	76.9	78.3	73.9	73.0	74.9	70.5	69.8	71.2	70.6	70.2	71.0	70.9	70.4	71.3	62.4	62.0	62.8	66.3	65.4	67.2	64.7	63.6	65.9	70.1	69.7	70.6
Flu	15.2	14.9	15.4	17.8	17.4	18.2	11.9	11.6	12.1	19.4	19.2	19.6	19.3	19.0	19.5	17.4	17.1	17.6	13.7	13.3	14.0	15.3	15.0	15.7	18.9	18.6	19.1
Hep A	98.3	97.6	99.1	96.9	95.8	97.9	92.8	92.0	93.6	93.6	93.1	94.1	93.8	93.3	94.3	82.8	82.3	83.3	86.7	85.5	88.0	75.8	74.4	77.2	94.4	93.9	94.9
IPV	80.3	79.6	81.0	76.6	75.6	77.5	73.1	72.4	73.8	72.9	72.5	73.4	73.2	72.8	73.6	64.5	64.0	64.9	69.4	68.3	70.5	65.2	64.0	66.3	72.7	72.3	73.2
Kindergarten	69.8	69.1	70.4	68.5	67.6	69.4	66.0	65.3	66.7	62.8	62.4	63.2	63.0	62.6	63.5	55.5	55.1	55.9	60.4	59.5	61.3	54.3	53.3	55.3	62.9	62.5	63.4
MMR	82.5	81.8	83.2	79.2	78.2	80.1	75.4	74.7	76.1	76.3	75.9	76.7	76.7	76.3	77.2	67.6	67.2	68.0	71.6	70.6	72.7	67.8	66.6	68.9	76.0	75.6	76.5
VAR	81.8	81.1	82.5	78.3	77.3	79.2	74.6	73.9	75.3	75.1	74.6	75.5	75.4	75.0	75.9	66.3	65.9	66.8	71.8	70.8	72.9	63.9	62.8	65.1	75.3	74.9	75.8
**Urban counties ^3^**	*P1*	*95% CI*		*P2*	*95% CI*		*P3*	*95% CI*		*Male*	*95% CI*		*Female*	*95% CI*		*White*	*95% CI*		*Black*	*95% CI*		*Hispanic*	*95% CI*		*Non-Hispanic*	*95% CI*	
DTaP	72.7	72.0	73.4	69.7	68.7	70.6	68.8	68.2	69.5	74.2	73.7	74.7	74.6	74.2	75.1	69.1	68.7	69.5	66.9	65.9	67.8	71.0	70.1	71.9	74.8	74.4	75.3
Flu	18.4	18.0	18.7	23.2	22.7	23.8	18.2	17.8	18.6	14.7	14.5	14.9	14.5	14.3	14.6	13.6	13.4	13.7	9.7	9.4	10.0	21.8	21.3	22.2	14.5	14.3	14.6
Hep A	94.5	93.7	95.3	94.2	93.1	95.3	92.3	91.6	93.1	96.0	95.4	96.5	96.4	95.9	96.8	89.4	88.9	89.9	89.3	88.0	90.5	81.8	80.9	82.7	97.4	96.9	97.9
IPV	75.0	74.3	75.7	72.0	71.1	72.9	71.2	70.5	71.8	76.9	76.4	77.3	77.3	76.8	77.8	71.7	71.2	72.1	70.9	69.9	72.0	70.4	69.5	71.2	77.7	77.2	78.1
Kindergarten	63.0	62.3	63.7	62.9	62.0	63.9	62.8	62.1	63.4	68.0	67.5	68.4	68.5	68.1	68.9	63.3	62.9	63.8	62.6	61.7	63.5	57.9	57.2	58.5	69.0	68.6	69.5
MMR	78.3	77.7	79.0	75.7	74.8	76.7	74.6	74.0	75.2	79.2	78.8	79.7	79.6	79.1	80.0	73.8	73.3	74.2	73.2	72.2	74.2	75.4	74.5	76.3	79.9	79.5	80.4
VAR	77.0	76.3	77.7	74.5	73.6	75.4	73.4	72.8	74.0	78.5	78.0	78.9	78.8	78.3	79.2	73.0	72.5	73.4	73.3	72.2	74.3	69.4	68.6	70.2	79.5	79.0	79.9

^1^ DTaP (Diphtheria, Tetanus, and Pertussis), Flu (Influenza), Hep A (Hepatitis A), IPV (Poliomyelitis), MMR (Measles, Mumps, and Rubella), VAR (Varicella/Chickenpox). Kindergarten Series was part of TennIIS vaccine coverage data collection for children within age group 4–6 years. This series is comprised of the DTaP, IPV, MMR, Hep A/B, and VAR, and focuses on the list of vaccines required for children before they enter Kindergarten. ^2^ Three COVID pandemic periods were created based on Tennessee’s COVID timeline as follows: Pre-pandemic period (P1: January 2017 to December 2019), stay-at-home period (P2: January 2020 to May 2021), and reopening period (P3: June 2021 to September 2023). ^3^ Rurality where each county was classified as either rural or urban was based on the 2023 USDA-RUCC.

## Data Availability

The datasets presented in this article are not readily available because the IRB stated that data cannot be shared publicly without a new IRB protocol application requesting the data from Tennessee Department of Health.
